# Supplementary material for: Risk factors for sacrococcygeal pilonidal sinus: a systematic review and meta-analysis supplemented by genetic causal assessment
Source: Front Surg. 2026 Jan 7;12:1718589. doi: 10.3389/fsurg.2025.1718589 (PMC12819706; doi:10.3389/fsurg.2025.1718589)
Supplement: Supplementary file 2 [file Datasheet2.zip › Supplementary Data 2/MR_pipeline_p5e-8/forest_plots/ukb-b-223.finngen_R12_L12_PILONIDALCYST.pdf]

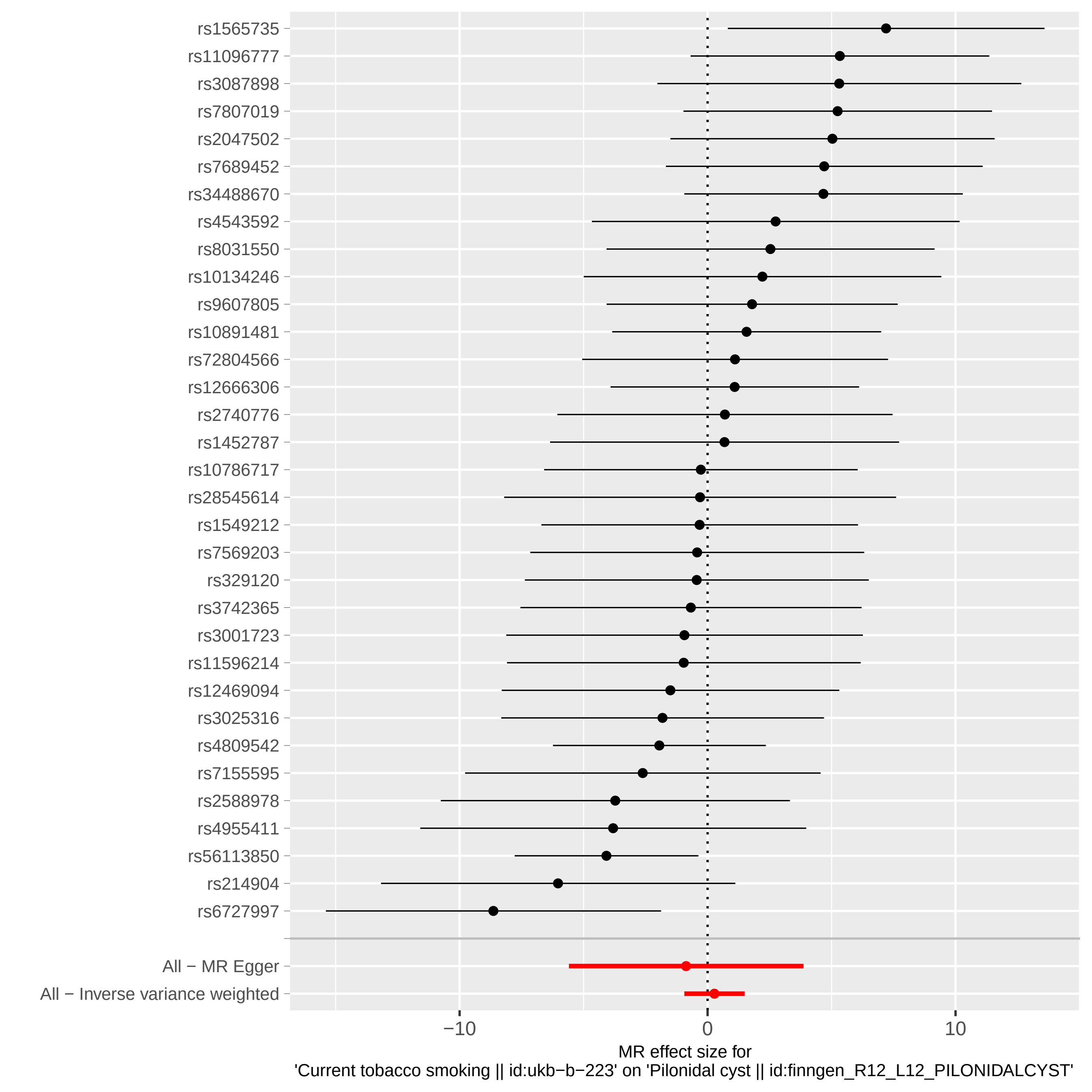

rs1565735
rs11096777
rs3087898
rs7807019
rs2047502
rs7689452
rs34488670
rs4543592
rs8031550
rs10134246
rs9607805
rs10891481
rs72804566
rs12666306
rs2740776
rs1452787
rs10786717
rs28545614
rs1549212
rs7569203
rs329120
rs3742365
rs3001723
rs11596214
rs12469094
rs3025316
rs4809542
rs7155595
rs2588978
rs4955411
rs56113850
rs214904
rs6727997
All − MR Egger
All − Inverse variance weighted
−10
0
10
MR effect size for
'Current tobacco smoking || id:ukb−b−223' on 'Pilonidal cyst || id:finngen_R12_L12_PILONIDALCYST'
